# Constructing Multiphase‐Induced Interfacial Polarization to Surpass Defect‐Induced Polarization in Multielement Sulfide Absorbers

**DOI:** 10.1002/advs.202307649

**Published:** 2023-12-03

**Authors:** Shengchong Hui, Xu Zhou, Limin Zhang, Hongjing Wu

**Affiliations:** ^1^ MOE Key Laboratory of Material Physics and Chemistry under Extraordinary School of Physical Science and Technology Northwestern Polytechnical University Xi'an 710072 P. R. China

**Keywords:** electromagnetic wave absorption, high‐entropy materials, interfacial polarization, multiphase interfacial engineering

## Abstract

The extremely weak heterointerface construction of high‐entropy materials (HEM) hinders them being the electromagnetic wave (EMW) absorbers with ideal properties. To address this issue, this study proposes multiphase interfacial engineering and results in a multiphase‐induced interfacial polarization loss in multielement sulfides. Through the selection of atoms with diverse reaction activities, the multiphase interfacial components of CuS (1 0 5), Fe_0.5_Ni_0.5_S_2_ (2 1 0), and CuFe_2_S_3_ (2 0 0) are constructed to enhance the interfacial polarization loss in multielement Cu‐based sulfides. Compared with single‐phase high‐entropy Zn‐based sulfides (ZnFeCoNiCr‐S), the multiphase Cu‐based sulfides (CuFeCoNiCr‐S) possess optimized EMW absorption properties (effective absorption bandwidth (EAB) of 6.70 GHz at 2.00 mm) due to the existence of specific interface of CuS (1 0 5)/CuFe_2_S_3_ (2 0 0) with proper EM parameters. Furthermore, single‐phase ZnFeCoNiCr‐S into FeNi_2_S_4_ (3 1 1)/(Zn, Fe)S (1 1 1) heterointerface through 400 °C heat‐treated is decomposed. The EMW absorption properties are enhanced by strong interfacial polarization (EAB of 4.83 GHz at 1.45 mm). This work reveals the reasons for the limited EMW absorption properties of high‐entropy sulfides and proposes multiphase interface engineering to improve charge accumulation and polarization between specific interfaces, leading to the enhanced EMW absorption properties.

## Graphical abstract

This work shows that the weak electron exchange effect induced by high conformational entropy adversely affects microwave absorption. Constructing the heterointerfaces with significantly different work functions in multi‐element sulfides can effectively enhance the phase interface polarization and eliminate these negative effects. This is expected to serve as a new design guideline for microwave absorbers.

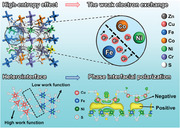

## 1 Introduction

High‐entropy materials (HEM), as a new type of concentrated solid solutions, have attracted great attention in the field of functional materials such as catalysis, energy, batteries, owing to their significant physicochemical properties.^[^ ^1^, ^2^, ^3^, ^4^, ^5^, ^6^, ^7^, ^8^, ^9^ ^]^ But as the research advances, more features are revealed about HEM, which encompasses a range of adverse consequences caused by high‐entropy strategy. For HEM containing large radius atoms, the electron spin channels encounter strong disorderly scattering, resulting in a decrease in electron transport.^[^ ^10^ ^]^ In addition, high configuration entropy may promote the formation of single‐phase solid solution rather than multi‐phase compounds, so that the interfacial construction of HEM is weakly reflected.^[^ ^11^ ^]^ In recent years, HEM have been preliminarily applied in the field of electromagnetic wave (EMW) absorption.^[^ ^12^, ^13^, ^14^, ^15^ ^]^ The EMW absorption mechanism of HEM has always been a hot research topic. Several researches have focused on high‐entropy‐induced dense defect sites that favor EMW absorption properties.^[^ ^16^, ^17^ ^]^ However, the contribution from the interface/conductivity/defect needs to be comprehensively considered to properly assess the dielectric loss capability, but existing investigations may neglect the potential hazards of high‐entropy strategy on interfaces. Hence, eliminating the adverse impact of weak heterointerfaces on EMW absorption properties in high‐entropy absorbers has emerged as a great scientific challenge.

To address the above challenge, the following research framework should be constructed in depth. 1) It is imperative to employ an effective design guideline that generates abundant heterointerfaces within the HEM. Additionally, it is necessary to prove the essential contribution of interfaces and explore the ideal range of electromagnetic (EM) parameters for effectively dissipating EMW energy. 2) Apart from the modulation of interfaces, it is equally crucial to maintain the numerous defect sites of the HEM to attain a significant density of polarization centers. 3) Although it was said above about design of heterointerfaces, it is impossible to maintain abundant heterointerfaces within the HEM due to its single‐phase solid solution characteristic.

Inspired by conventional interface engineering,^[^ ^18^, ^19^, ^20^, ^21^, ^22^, ^23^, ^24^ ^]^ a novel multiphase interfacial engineering provides a possible approach to implement the above research framework. Specifically, the stronger reaction activity provides certain atoms with significant chemical reaction advantages, leading to the preferential formation of specific chemical components during the reaction process, and impeding the attempts of less activity atoms to substitute within the original lattice. While for atoms with comparable reaction activity, the nucleation phenomena occur almost simultaneously, resulting in a highly random and compatible arrangement of these atoms in the lattice. Hence, by carefully selecting the types and quantities of reacting atoms, it becomes feasible to attain effective manipulation over the interface components as well as the configuration entropy of individual components. Transition metal sulfides (TMSs) were selected as substrates due to the low electronegativity of S, resulting in a weaker metal─sulfur (M─S) bond that may enhance the probability of phase separation. Benefitting from the differences in the reaction activity of atoms, after adding the synergistic elements (Fe, Co, Ni, and Cr), CuS tends to form multiphase compounds, while ZnS is more likely to form single‐phase high‐entropy solid solution, as postulated by the Hume–Rothery rule (smaller sulfur bond length favors single‐phase stability).^[^ ^25^, ^26^, ^27^ ^]^ The interfaces and configuration entropy of Cu‐based sulfides can be modified by specific combinations of elements. Multiple synergistic elements experience random substitution within various lattices to form several medium‐entropy sulfides, thereby preserving a significant number of defect sites.

Herein, we proposed a novel multiphase interfacial engineering to eliminate the adverse impact of weak heterointerfaces on the EMW absorption properties in high‐entropy sulfides. Stable high‐entropy Zn‐based sulfides and phase‐separated Cu‐based sulfides were synthesized by a facile solvothermal approach. The results show that the high configuration entropy will result in a decrease in the charge density at Co atom sites and its weak electron exchange effect between Co/Ni and Fe atoms in high‐entropy Zn‐based sulfides, leading to the lower EM parameters. As expected, the high‐entropy Zn‐based sulfides (ZnFeCoNiCr‐S) were failing to effectively absorb EMW due to their limited heterointerface effects and low complex permittivity. In contrast, the multiphase Cu‐based sulfides (CuFeCoNiCr‐S) showed satisfactory EMW absorption properties (i.e., EAB: 6.70 GHz at 2.00 mm) owing to the multiphase‐induced interfacial polarization loss, especially caused by the specific interface of CuS (1 0 5)/CuFe_2_S_3_ (2 0 0). Furthermore, the single‐phase ZnFeCoNiCr‐S was transformed into a phase‐separation structure through thermal decomposition at 400 °C. Due to the strong interfacial polarization induced by the FeNi_2_S_4_ (3 1 1)/(Zn, Fe)S (1 1 1) phase interfaces, the EMW absorption properties are significantly enhanced (EAB of 4.83 GHz at 1.45 mm). This study reveals the reasons for the limited EMW absorption properties of high‐entropy sulfides and proposes multiphase interface engineering strategies to enhance interfacial charge accumulation and polarization, and achieves optimization of EMW absorption properties.

## 2 Results and Discussion

### 2.1 Synthesis and Phase Analysis of Zn‐Based and Cu‐Based Sulfides

**Figure 1:**
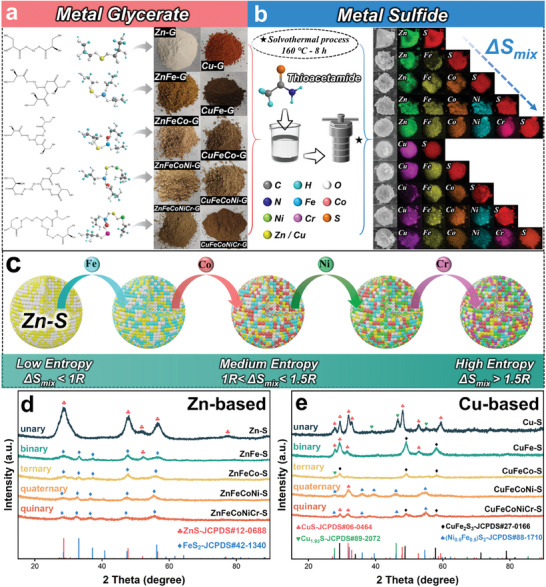
Synthesis and phase analysis of Zn‐based and Cu‐based sulfides. a) Molecular structure and macroscopic morphology of metallic glyceride precursors. b) Microscopic morphology and EDS mapping images of Zn‐based and Cu‐based sulfides. c) Increased mixing entropy driving Zn‐based sulfides to form high‐entropy‐stabilized structure. Powder XRD pattern of d) Zn‐based sulfides and e) Cu‐based sulfides.

The reported method has been used to synthesize a variety of metallic glycerate precursors as intermediate templates (**Figure** **1**a), which are almost amorphous structures at this stage.^[^ ^28^, ^29^ ^]^ After facile solvothermal sulfidation, various metal elements are confined in the same micrometer‐sized particle. Finally, we successfully prepare the multi‐principal Zn‐based/Cu‐based sulfides and their subset species (Figure S1–S3, Supporting Information). The energy dispersive spectroscopy (EDS) mapping was used to clarify the local chemical composition, and the result shows that all elements are uniformly distributed without significant accumulation or separation of any specific element (Figure 1b). High‐entropy sulfides should be single‐phase stability, because compatible atoms replace the original lattice sites, leading to a continuous increase in the mixing entropy^[^ ^30^ ^]^ (Figure 1c). In order to assess whether the samples have single‐phase stable structure, X‐ray diffraction (XRD) was employed to obtain crystal structure information for all samples. As shown in Figure 1d and Table S1 (Supporting Information), it is evident that the binary sulfide (ZnFe‐S) exhibits a second crystal structure signal, which is eliminated as the principal elements increase (unary: hexagonal system → binary: hexagonal system/face‐centered cubic [FCC] → ternary/quaternary/quinary: FCC), indicating the stability of the phase structure in Zn‐based sulfides is controlled by the increasing mixing entropy. In contrast, Figure 1e and Table S2 (Supporting Information) illustrate that phase separation is observed in the Cu‐based sulfides (from unary to quaternary: bi‐phases, quinary: tri‐phases). The slight angular deviation of the diffraction peaks serves as evidence that the specific elements are not present as new phases, but rather as substitutions of cation sites in the crystalline. Furthermore, there is a notable reduction in the intensity of the diffraction peaks, which can be attributed to the uneven Bragg surfaces due to the atomic disorder substitution.^[^ ^31^ ^]^ These findings suggest that the multi‐principal Cu‐based sulfides are compounded by several medium‐entropy phases. Based on the phase composition of samples, we have summarized the reaction activity order in our system as follows: high‐activity: Cu, medium activity: Fe≈ Co≈ Ni> Zn, low activity: Cr. In short, we have accomplished the fabrication of the multiphase interfacial engineering via a facile synthetic route. It can be inferred that the multi‐principal Cu‐based sulfides not only provide rich heterointerfaces, but also retain numerous defect sites due to these medium‐entropy phases.

### 2.2 The Effect and Mechanism of High‐Entropy Strategy on the EMW Absorption Properties of Zn‐Based Sulfides

**Figure 2:**
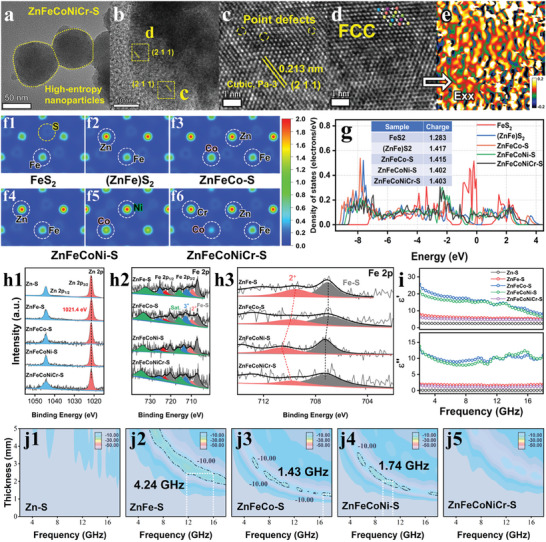
The microscopic characterization, simulation calculation, and electromagnetic wave absorption properties of Zn‐based sulfides. a–d) HR‐TEM images of ZnFeCoNiCr‐S sample. e) The strain distribution image of ZnFeCoNiCr‐S sample. Charge density images of f1) FeS_2_, f2) (ZnFe)S_2_, f3) ZnFeCo‐S, f4,f5) ZnFeCoNi‐S, f6) ZnFeCoNiCr‐S. g) The density of states of Zn‐based sulfides. High‐resolution XPS spectra of Zn‐based sulfides, h1) Zn element, h2,h3) Fe element. i) The electromagnetic parameters of Zn‐based sulfides. j1,j5) The electromagnetic wave absorption properties of Zn‐based sulfides.

To investigate the accurate lattice configurations of high‐entropy Zn‐based sulfide at nanoscale, the ZnFeCoNiCr‐S sample was characterized by high‐resolution transmission electron microscopy (HR‐TEM) (Figure S4, Supporting Information). As shown in **Figure** **2**a–d, the high‐entropy nanoparticles expose interplanar spacing of 0.213 nm, which is attributed to (2 1 1) faces of the space group Pa‐3 cubic. Geometric phase analysis (GPA) technology was employed to assess the local strain distribution in ZnFeCoNiCr‐S sample, where the positive and negative values represent the lattice in tension and compression respectively. After applying a stress field in the *E*_xx_ direction, it is clearly observed that the lattice possesses high strain gradient, indicating that severe distortion occurs in the lattice^[^ ^32^, ^33^ ^]^ (Figure 2e). Furthermore, in order to elucidate the regulation of the charge state with increasing configuration entropy in Zn‐based sulfides, we have calculated the charge distribution and density of states (DOS) based on density flooding theory (DFT).^[^ ^34^ ^]^ Figure 2f1–f6 and Figure S5 (Supporting Information) give the charge distribution in the FCC structure from low‐entropy to high‐entropy sulfides. The results show that as the configuration entropy gradually increases, there is no obvious charge accumulation or separation in the nearby areas of all metal atoms. This implies that the value of configuration entropy in the FCC structure has little influence on the nearby charge distribution of the metal atoms. In addition, we can observe an increase in the charge density at Co atomic sites in quaternary sulfide (ZnFeCoNi‐S) compared to ternary sulfide (ZnFeCo‐S). Nonetheless, with the addition of a fifth metal element (ZnFeCoNiCr‐S), the charge density at Co atomic sites sharply decreases, indicating that high configuration weakens the electron exchange process. Next, as shown in Figure 2g, the DOS image provides the relative number of electrons in the specified energy range from low‐entropy to high‐entropy FCC structure. The integral area of DOS is positively correlated with the number of electrons present in the energy band.^[^ ^35^ ^]^ The results reveal a sharp upsurge in the area only for the unary to binary FCC structure (from 1.283 to 1.417), while the change is negligible for the binary to quinary FCC structure (1.417→ 1.415→ 1.402→ 1.403). Thus, it can be inferred that the high configuration entropy has little impact on both the quantity of electrons and the localization pattern in the FCC structure.

In order to accurately clarify the electron exchange behavior of the atoms in Zn‐based sulfides, X‐ray photoelectron spectroscopy (XPS) was carried out to measure the chemical states of the elements. The fitting curves show that the peak binding energy corresponding to Zn element remains constant (1021.4 eV) with increasing configuration entropy, indicating that Zn atoms exhibit little involvement in electron exchange processes (Figure 2h1). Compared to ZnFe‐S sample, it is observed that the feature peak signal of Fe(II) gradually shifted toward higher binding energy (Fe^2+^→Fe^δ+^, 2< δ< 3) in the ZnFeCo‐S and ZnFeCoNi‐S samples, but the high configuration entropy (ZnFeCoNiCr‐S) resulted in a smaller shift (Figure 2h2,h3). As shown in Figure S6 (Supporting Information), based on the convolution results of Co 2p and Ni 2p, we can infer that Fe atoms serve as electron donor and Co/Ni atoms act as electron acceptors during the electron exchange process. These results demonstrate that the medium‐entropy FCC structure significantly facilitates the electron exchange process, while the high‐entropy FCC structure may weaken this process, agreeing with the DFT calculation results. In general, the atoms in the lattice can form chemical bonds via electron exchange. When exposure to an external electromagnetic field, the electrons will be repositioned, leading to fluctuation in the complex permittivity of materials.^[^ ^36^ ^]^ Figure 2i and Figure S7 (Supporting Information) provide the EM parameters (*ε*′ and *ε*″; *μ*′ and *μ*″) of Zn‐based sulfides with the filler loading 50 wt.%. As expected, with the configuration entropy increasing, the complex permittivity (*ε*′ and *ε*″) of samples exhibits the regulation of an initial increase (from unary to quaternary) followed by a decline (quinary). The reflection loss (RL) of Zn‐based sulfides was calculated to evaluate the EMW absorption properties (Figure 2j1–j5; Table S3, Supporting Information). The results indicate that the high‐entropy sulfides (ZnFeCoNiCr‐S) exhibit poor EMW absorption properties, while the medium‐entropy sulfides (ZnFe‐S, ZnFeCo‐S, ZnFeCoNi‐S) can absorb more than 90% EMW energy in specific frequency range. The binary sulfide (ZnFe‐S) possesses the best EMW absorption properties, where effective absorption bandwidth with RL≤ −10 dB is 4.24 GHz at 2.2 mm.

To better understand the EMW absorption mechanisms of Zn‐based sulfides in our model, the following detailed discussion should be carried out. First, Figure S8 (Supporting Information) shows the permittivity tangent (tan δ_ε_) and the permeability tangent (tan δ_μ_) of absorbers to explore the dominant loss mechanism. It is clear that the value and variation of tan δ_μ_ are much smaller than that of tan δ_ε_, indicating that the magnetic loss only plays a minor role, while EMW absorbers are dominated by dielectric loss. Second, electrochemical impedance spectroscopy (EIS) was employed to measure the charge transfer resistance (*R*_ct_) for assessing the contribution of the conductive loss, where a smaller semicircle implies a lower resistance and a higher conduction^[^ ^37^, ^38^, ^39^ ^]^ (Figure S9, Supporting Information). The results show that the intrinsic *R*_ct_ of Zn‐based sulfides is relatively high (>10^4^ Ω). The excessive energy barrier limits the electrons hopping, consequently impeding the formation of conductive network. Thus, for Zn‐based sulfides, we can consider that the conductive loss does not play a dominate role in the EMW absorption process. Third, in high‐entropy FCC structure, the differences of atomic sizes will lead to serious lattice distortion (Figure 2e). Therefore, each atom may hinder the transform of free electrons, thereby weakening the strength of electron exchange (Figure 2f–h) and resulting in a low complex permittivity (Figure 2i). A low complex permittivity indicates that the absorber is less responsive to EM fields, resulting in weak defect‐induced polarization behavior. Fourth, considering that the binary Zn‐based sulfide (ZnFe‐S) is a compound of ZnS and FeS_2_ (Figure S10, Supporting Information), which results in the generation of numerous heterointerfaces, leading to the charge accumulation and subsequent interfacial polarization loss. The interfacial polarization effect has been widely used to promote EMW absorption properties.^[^ ^40^ ^]^ In short, the poor EMW absorption properties of high‐entropy Zn‐based sulfide can mainly be explained by the low complex permittivity induced by the reduced electron exchange effect in high‐entropy FCC structure and the weak interfacial polarization loss due to its single‐phase high‐entropy solid solution.

### 2.3 The Effect and Mechanism of Multiphase Interfacial Engineering on the EMW Absorption Properties of Cu‐Based Sulfides

**Figure 3:**
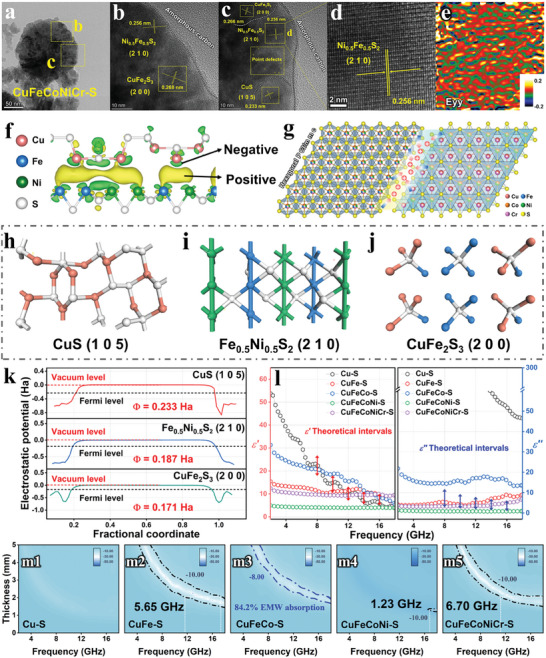
The microscopic characterization, first‐principle calculations, and electromagnetic wave absorption properties of Cu‐based sulfides. a–d) HR‐TEM images of CuFeCoNiCr‐S sample. e) The strain distribution image of CuFeCoNiCr‐S sample. f) The differential charge density mapping of CuS/Fe_0.5_Ni_0.5_S_2_ heterointerface. g) The polarization caused by charge accumulation at the interfaces. The computational models corresponding to the faces of h) CuS (1 0 5), i) Fe_0.5_Ni_0.5_S_2_ (2 1 0), and j) CuFe_2_S_3_ (2 0 0). k) First‐principle calculations for the work function of three models. l) The electromagnetic parameters and theoretical effective absorption intervals of Cu‐based sulfides. m1–m5) The electromagnetic wave absorption properties of Cu‐based sulfides.

In contrast to Zn‐based sulfides, multi‐principal Cu‐based sulfides possess refined heterointerface structure, which is regulated by the reaction activity of metal atoms. The HR‐TEM images were utilized to gain the information about the grain boundary distribution of quinary Cu‐based sulfides (CuFeCoNiCr‐S) with various phases, as shown in **Figure** **3**a–d and Figure S11 (Supporting Information). The CuFeCoNiCr‐S sample shows typical phase‐separation structure, accompanied by numerous heterointerfaces, including the (2 0 0) face of CuFe_2_S_3_, the (2 1 0) face of Fe_0.5_Ni_0.5_S_2_ and the (1 0 5) face of CuS. The lattice strain was characterized by the GPA technology (Figure 3e), and the corresponding strain mapping indicates that the presence of severe lattice distortion in CuFeCoNiCr‐S. This retained lattice distortion phenomenon can be attributed to the substitution of compatible atoms for lattice sites, indicating the medium configuration entropy of various components in CuFeCoNiCr‐S sample.^[^ ^41^ ^]^ The charge transfer state of the heterointerface models was simulated by first‐principle calculations to emphasizes the interfacial effect in Cu‐based sulfides. Specifically, heterojunction was constructed by utilizing CuS and Fe_0.5_Ni_0.5_S_2_ as models. The differential charge density mapping (Figure 3f) reveals that positive charge primarily gathered in the heterointerface. As shown in Figure 3g, the local accumulation of space charges forms significant interfacial polarization, which enhances the EMW absorption properties of Cu‐based sulfides. Typically, the work function that represents the energy for removing a charge was employed to evaluate the strength of interfacial polarization.^[^ ^42^ ^]^ Based on the heterointerfaces observed by HR‐TEM, we have constructed three computational models (Figure 3h–j) corresponding to the faces of CuS (1 0 5), Fe_0.5_Ni_0.5_S_2_ (2 1 0) and CuFe_2_S_3_ (2 0 0), respectively. Next, we calculated the work functions for these models using the DFT as theoretical basis. Figure 3k and Figure S12 (Supporting Information) presents the difference in the work function of three models, suggesting that the interfacial polarization occurs in the Cu‐based sulfides and that the polarization strength is ordered as follows: CuS (1 0 5)/CuFe_2_S_3_ (2 0 0) > CuS (1 0 5)/Fe_0.5_Ni_0.5_S_2_ (2 1 0) > CuFe_2_S_3_ (2 0 0)/Fe_0.5_Ni_0.5_S_2_ (2 1 0). To explore the reasons for the differences in interfacial polarization strengths, we calculated the bandgap of CuS, CuFe_2_S_3_, and Fe_0.5_Ni_0.5_S, respectively (Figure S13, Supporting Information). The bandgap, which reflects the efficiency of charge transfer, exhibits marked differences between CuS and both CuFe_2_S_3_ and Fe_0.5_Ni_0.5_S_2_. In contrast, the differences in bandgap width between CuFe_2_S_3_ and Fe_0.5_Ni_0.5_S_2_ are relatively small. Hence, the significant interfacial polarization fundamentally relies on the differences in charge transfer ability of components.

Figure 3l and Figure S14 (Supporting Information) exhibit the EM parameters of Cu‐based sulfides to facilitate a comprehensive understanding of the absorber's EM response behavior. From the complex permittivity curves, it is hard to summarize the variation pattern only based on the values, because the complex permittivity is controlled by the synergistic contributions of the phase‐separation components. In order to assess the merits of EM parameters, we predicted the range of complex permittivity that satisfy the ideal absorption properties (RL < −10 dB) at specified frequency points,^[^ ^43^ ^]^ Figure S15 (Supporting Information). All of the predictions are based on a given permeability (*μ*′ = 1 and *μ*″ = 0) and thickness (*d* = 2.00 mm), and the results have been marked with arrows. The results demonstrate that the EM parameters of CuFe‐S and CuFeCoNiCr‐S samples at high‐frequency range (12–18 GHz) have a good match with the theoretical interval. Thus, it can be inferred that CuFe‐S and CuFeCoNiCr‐S may possess superior EMW absorption properties compared to other Cu‐based sulfides. The measured 2D RL images further supported the above viewpoints (Figure 3m1–m5; Table S4, Supporting Information). As expected, the CuFe‐S and CuFeCoNiCr‐S samples achieved excellent EMW absorption properties at high‐frequency, with effective absorption bandwidth of 5.65 and 6.70 GHz, respectively. The impedance mismatch caused by too high EM parameters of Cu─S, resulting in the poor EMW absorption properties (Figure S16, Supporting Information). Furthermore, the CuFeCo‐S and CuFeCoNi‐S samples exhibit significantly enhanced EMW absorption properties compared to Cu─S sample, with the ability to absorb 84.2% or even 90% EMW energy in a specific range. These findings indicate that the multiphase interfacial engineering is an effective strategy for optimizing EMW absorption properties of Cu‐based sulfides.

Similarly, a more detailed discussion needs to be carried out to reveal the EMW loss mechanisms of Cu‐based sulfides. The very low permeability of Cu‐based sulfides suggests that we can ignore the impact of magnetic loss (Figure S17, Supporting Information). The EIS fitting results show large *R*_ct_ (>10^4^ Ω) for the absorbers (Figure S18, Supporting Information), suggesting that the conductive loss does not play a dominant role in our absorbers. Subsequently, we have organized the theoretical calculation data to achieve a comprehensive understanding of the loss mechanism and the corresponding influence factors. First, the differential charge density map calculated by first‐principle calculations indicates that the accumulation of numerous charges at the heterointerface, triggering the interfacial polarization behavior (Figure 3f). Second, the interfacial polarization strength of different heterojunctions exhibits obvious variations (Figure 3k; Figure S12, Supporting Information). The calculated work function indicates that the order of polarization strength is CuS (1 0 5)/CuFe_2_S_3_ (2 0 0) > CuS (1 0 5)/Fe_0.5_Ni_0.5_S_2_ (2 1 0) > CuFe_2_S_3_ (2 0 0)/ Fe_0.5_Ni_0.5_S_2_ (2 1 0). For the samples with strongly polarized heterojunction (e.g., CuFeCoNiCr‐S, CuFe‐S, Figure 1e), the EMW absorption properties have been significantly optimized. The above results indicate that multiphase‐induced interfacial polarization plays a dominant role in the EMW absorption process. Third, the band structure reveals that the strengths of polarization fundamentally arise from the variations in the charge transfer capability of interfacial components (Figure S13, Supporting Information). Therefore, in the design of heterointerface, we should adhere to this guideline and select ideal reaction species combinations in the huge materials database based on the reaction activity of atoms. Fourth, in the multielement Cu‐based sulfides, it seems that the multiphase interfaces such as CuS (1 0 5)/CuFe_2_S_3_ (2 0 0)/Fe_0.5_Ni_0.5_S_2_ (2 1 0), especially the specific interface of CuS (1 0 5)/CuFe_2_S_3_ (2 0 0), are very important for multiphase‐induced interfacial polarization to improve the EMW absorption properties of Cu‐based sulfides (Figure 3m).

### 2.4 Constructing Strong Phase Interfacial Polarization to Enhance the EMW Absorption Properties of Multielement Zn‐Based Sulfides

**Figure 4:**
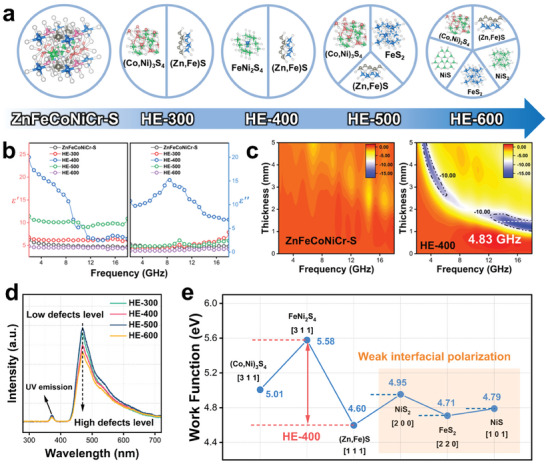
a) The XRD pattern of HE‐300, HE‐400, HE‐500 and HE‐600. b) The EM parameters of HE‐300, HE‐400, HE‐500 and HE‐600. c) 2D‐RL plot of HE‐400 sample. d) The measured PL spectra of samples. e) Work functions of different phase interfaces.

The above research focuses on elucidating the effectiveness of multiphase interfaces in improving EMW absorption properties, but fundamentally there is still a lack of direct comparison between high‐entropy and multiphase absorbers within the same elemental species. Therefore, we decompose single‐phase high‐entropy sulfide (ZnFeCoNiCr‐S) into multiphase through a heat‐treated approach in an inert gas atmosphere, with the aim of achieving effective interface construction. Since the atomic motion and the nucleation/crystallization processes are controlled by the thermodynamic temperature, the crystal structure can be influenced by changing the annealing temperature. As shown in **Figure** **4**a, at different annealing temperatures (300–600 °C, named HE‐300, HE‐400, HE‐500, and HE‐600, respectively), complex self‐reconstruction processes occur in ZnFeCoNiCr‐S samples, leading to differences in phase composition. From HE‐300 to HE‐400, the crystal structure and space group of the sample did not change (both are Cubic, Fd‐3m and Cubic F‐43m), but the XRD patterns (Figure S19, Supporting Information) show the shift of the diffraction peak that indicates atomic substitution (i.e., in the HE‐400 sample, Co atoms replaced some Fe atoms, resulting in the diffraction peak of Cubic, Fd‐3m shifted toward a small angle). As the temperature continued to increase, the number of phases in the samples increased, indicating the generation of more heterogeneous interfaces (HE‐500: (Co, Ni)_3_S_4_/(Zn, Fe)S/FeS_2_, HE‐600: (Co, Ni)_3_S_4_/(Zn, Fe)S/FeS_2_/NiS/NiS_2_).

To investigate the EM response behavior, the EM parameters of all samples were measured in 2–18 GHz with the same filler loading of 50 wt.% (Figure 4b). As the annealing temperature increases, we can clearly observe that the *ε*’ and *ε*’’ exhibit pattern of first increasing and then decreasing, reaching the highest value in HE‐400. This suggests the strong response and attenuation behavior of the incident EMW with HE‐400. From the dielectric curves of HE‐400 and HE‐500, the significant relaxation peaks indicate that the polarization loss may occur inside the absorbers. In addition, only the dielectric curve in high frequency of the HE‐400 sample is close to the theoretical interval that satisfy the ideal EMW absorption properties (Figure S15, Supporting Information). Hence, we can infer that the HE‐400 may possess remarkable enhanced EMW absorption properties, while HE‐300, HE‐500, and HE‐600 cannot achieve broadband EMW absorption. Figure 4c and Figure S20 (Supporting Information) exhibit the 2D‐RL plots of all samples, as expected, due to its superior dielectric properties and relaxation behavior, the EAB of HE‐400 reaches 4.83 GHz at a thickness of 1.45 mm. Even though HE‐500 and HE‐600 have more phase interfaces, their EMW absorption properties are inferior to HE‐400 (HE‐500: 1.31 GHz, 2.25 mm; HE‐600: 0 GHz).

In order to better understand the mechanisms of the above phenomena, it is imperative to carry out the following discussion. First, as shown in Figure S21 (Supporting Information), it is obvious that the values of *μ*’ and *μ*’’ fluctuate slightly ≈1 and 0, respectively. This indicates that magnetic loss is not sufficient to effectively dissipate EMW energy in our system, so the magnetic loss is not the dominant loss mechanism. Second, all samples are composed of semiconductor sulfides, accompanied by disordered atomic substitution, indicating that the conductive network inside the crystal is insufficient to form conduction loss. Therefore, we can eliminate the contribution of conduction loss in our samples. The extremely low *ε*’’ of HE‐300, HE‐500, and HE‐600 also support the above viewpoint. While the higher *ε*’’ of HE‐400 can be attributed to other relaxation polarization processes. Third, the crystallinity of the sample is influenced by the annealing temperature, thus modifying the defect level. The defect level tends to decrease as the annealing temperature increases in principle. Nevertheless, the formation of phase interfaces can damage the integrity of crystals, leading to an increase in the defect level. As a result, it is necessary to examine the defects present in the annealed sample in order to assess the impact of defect‐induced polarization. As shown in Figure 4d, photoluminescence (PL) spectra were applied to explore the excited states of electrons and holes. The peak at the 375 nm wavelength is attributed to the weak ultraviolet emission induced by the band edge excitons of the sample, indicating the presence of defects in the crystal. Usually, the higher the peak intensity at a wavelength of 470 nm, the lower the level of defects in the crystal. We can clearly find that the order of defect levels is: HE‐600>HE‐400>HE‐300>HE‐500, while the highest defect level does not enable HE‐600 to effectively absorb EMW. Therefore, in our system, defect‐induced polarization loss can be considered as not the dominant loss mechanism. Fourth, following the similar approach in Section 2.3, we evaluate the intensity of phase interfacial polarization by the value of the interface work function corresponding to the highest XRD diffraction peak. Figure 4e exhibits six‐phase interface work functions of heat‐treated samples. Among them, FeNi_2_S_4_ (3 1 1) and (Zn, Fe)S (1 1 1) possess the largest difference in work function (0.98 eV). Therefore, the superior EMW absorption properties of HE‐400 samples can be explained by the strong phase interfacial polarization. Compared to the two interfaces mentioned above, the difference in interface work functions between (Co, Ni)_3_S_4_ (3 1 1) and (Zn, Fe)S (1 1 1) is slightly smaller (0.41 eV), corresponding to moderate interface polarization. This explains why HE‐300 and HE‐500 can absorb over 90% of EMW in some frequency bands, even if their bandwidth is narrow. For the three‐phase interfaces of NiS_2_ (2 0 0), FeS_2_ (2 2 0), and NiS (1 0 1), the extremely low work function difference suggests that these phase interfaces are difficult to achieve charge accumulation and interfacial polarization. Even though HE‐600 has so many phase interfaces, its EMW absorption properties are very poor due to the weak interfacial polarization. This is consistent with the conclusion in Section 2.2 and 2.3. Therefore, in our system, the effect of phase interfacial polarization on EMW attenuation has surpassed defect‐induced polarization, and can be considered as the dominant loss mechanism.

## 3 Conclusion

In summary, the multiphase interfacial engineering was designed to eliminate the adverse impact of weak interface effect and low complex permittivity on the EMW absorption properties in high‐entropy sulfides. The different reaction activities of metal atoms promote the construction of heterointerfaces with pronounced differences in charge transfer ability in multielement Cu‐based sulfides. The EM parameters of Cu‐based sulfides fluctuate at 2–18 GHz due to the transition of phase among CuS/CuFe_2_S_3_/Fe_0.5_Ni_0.5_S_2_, while the low complex permittivity is induced by the reduced electron exchange effect in high‐entropy FCC structure. The multiphase‐induced interfacial polarization loss caused by the charge accumulation at the specific interface of CuS (1 0 5)/CuFe_2_S_3_ (2 0 0) optimize the EMW absorption properties of Cu‐based sulfides. Therefore, the multielement Cu‐based sulfides (CuFeCoNiCr‐S) achieves an EAB of 6.70 GHz at 2.00 mm. Furthermore, after heat‐treated at 400 °C, the single‐phase Zn‐based sulfide (ZnFeCoNiCr‐S) transforms into phase separation structure (HE‐400). The strong phase interfacial polarization of FeNi_2_S_4_ (3 1 1)/(Zn, Fe)S (1 1 1) leads to enhanced EMW absorption properties of multielement Zn‐based sulfide (EAB of 4.83 GHz at 1.45 mm). This work clarifies the reasons for the limited EMW absorption properties of high‐entropy sulfides, and proposes a multiphase interface engineering to improve interfacial charge accumulation and polarization behavior, and achieves enhanced EMW absorption properties in multielement sulfides.

## Conflict of Interest

The authors declare no conflict of interest.

## Supporting information

Supporting Information

## Data Availability

The data that support the findings of this study are available from the corresponding author upon reasonable request.
