# Supplementary material for: Constructing Multiphase‐Induced Interfacial Polarization to Surpass Defect‐Induced Polarization in Multielement Sulfide Absorbers
Source: Adv Sci (Weinh). 2023 Dec 3;11(6):2307649. doi: 10.1002/advs.202307649 (PMC10853738; doi:10.1002/advs.202307649)
Supplement: Supplementary file 1 — Supporting Information [file ADVS-11-2307649-s001.pdf]

ADVANCED SCIENCE
Open Access

Supporting Information

for *Adv. Sci.*, DOI 10.1002/advs.202307649

Constructing Multiphase-Induced Interfacial Polarization to Surpass Defect-Induced Polarization in Multielement Sulfide Absorbers

Shengchong Hui, Xu Zhou, Limin Zhang and Hongjing Wu**

Supporting Information

Constructing multiphase-induced interfacial polarization to surpass defect-induced polarization in multielement sulfide absorbers

Shengchong Hui, Xu Zhou, Limin Zhang *, Hongjing Wu *

MOE Key Laboratory of Material Physics and Chemistry under Extraordinary, School of Physical Science and Technology, Northwestern Polytechnical University, Xi'an 710072, P.R. China;

* Corresponding authors: liminzhang@nwpu.edu.cn (L. Zhang); wuhongjing@nwpu.edu.cn (H. Wu).

Experimental section

Raw Materials

Zinc nitrate, hexahydrate ($Zn(NO_3)_2.6H_2O$), Copper(II) nitrate, trihydrate ($Cu(NO_3)_2.3H_2O$), Iron(III) nitrate nonahydrate ($Fe(NO_3)_3.9H_2O$), Cobalt(II) nitrate hexahydrate ($Co(NO_3)_2.6H_2O$), Nickel(II) nitrate hexahydrate ($Ni(NO_3)_2.6H_2O$), chromium(III) nitrate nonahydrate ($Cr(NO_3)_3.9H_2O$), Thioacetamide (TAA, C_2H_5NS), Isopropyl alcohol (IPA, C_3H_8O), Glycerol ($C_3H_8O_3$), Anhydrous ethanol (C_2H_5OH) and deionized water were purchased from Aladdin Industrial Corporation (Shanghai, China). All the chemical reagents were used as received without further purification.

Synthesis of Zn-based sulfide and Cu-based sulfide

The entire reaction is carried out in two steps. **Firstly**, high-entropy glycerate templates were first synthesized using a facile solvothermal process. Specifically, equimolar (2 mmol each) metal nitrates of $Zn(NO_3)_2.6H_2O$, $Fe(NO_3)_3.9H_2O$, $Co(NO_3)_2.6H_2O$, $Ni(NO_3)_2.6H_2O$, $Cr(NO_3)_3.9H_2O$ were

dissolved in 50 mL IPA, followed by adding 10 mL glycerol. After thorough mixing, the mixed solution was transferred to a 100 mL Teflon-lined stainless-steel autoclave for solvothermal reaction at 150 °C for 10 h. The resulting powders were collected and used as the templates for the high-entropy metal sulfides synthesis. **Secondly**, 100 mg of the as-synthesized metal glycerate was dispersed in 50 mL of anhydrous ethanol, followed by the addition of 188 mg of thioacetamide. The mixed solution was then stirred for 30 min before transferring to a 100 mL Teflon-lined stainless-steel autoclave for the solvothermal synthesis at 160 °C for 8 h. The product (ZnFeCoNiCr-S) was centrifuged and washed three times with anhydrous ethanol and then dried at 60 °C for 48 h in a vacuum oven. Controlling samples of single metal sulfides (Zn-S and Cu-S), binary metal sulfides (ZnFe-S, CuFe-S), ternary metal sulfide (ZnFeCo-S, CuFeCo-S), quaternary metal sulfide (ZnFeCoNi-S, CuFeCoNi-S), and quinary metal sulfides (CuFeCoNiCr-S) were prepared using the same method expect using the corresponding as-synthesized metal glycerate templates.

Characterization methods

Materials Characterization

The crystalline structure and phase of prepared samples were characterized by X-ray diffraction (XRD, Cu Kα radiation) with a scanning range of $5° < 2\theta < 90°$. X-ray photoelectron spectroscopy (XPS, Kratos Axis Ultra DLD, UK) were employed on chemical state characterization. The scanning electron microscopy and energy dispersive spectra (SEM and EDS, ZEISS Sigma 300) were carried out to observe the microscopic surface morphology and the element distribution state of samples. The high-resolution transmission electron microscopy (HR-TEM, FEI Talos F200x) was employed to analysis the accurate lattice configurations of samples. The CHI 660E electrochemical workstation

was employed on EIS for analyzing the charge transmission resistance (R_{ct}). Specifically, a saturated calomel electrode, a Pt foil, and 1 M Na_2SO_4 aqueous solution was employed as reference electrode, counter electrode, and electrolyte, respectively.

Electromagnetic Measurement

The electromagnetic parameters of all samples were measured by the network vector analyzer (NVA, Anritsu MS46322B, Japan) in the 2-18 GHz frequency range. In this process, the sample and paraffin are uniformly mixed in a 50% mass ratio for using in coaxial testing (d_{out} = 7.00 mm, d_{in} = 3.04 mm). Typically, the reflection loss (RL) is used to evaluate the EMW absorption properties of absorbers. If the value of RL less than -10 dB (means 90% energy absorption), then the EMW absorption at that frequency point is considered effective. According the transmission line theory, RL was calculated by the following formula:

$$RL = 20\,log\left|\frac{Z_{in}-Z_0}{Z_{in}+Z_0}\right| \quad (1)$$

$$Z_{in} = Z_0\sqrt{\frac{\mu_r}{\varepsilon_r}}\,tanh\left(j\frac{2\pi fd}{c}\sqrt{\mu_r\varepsilon_r}\right) \quad (2)$$

Density functional theory (DFT) calculation

The differential charge density, density of states and work function calculation for the samples was carried out by the Vienna Ab initio Simulation Package (VASP) based on the Density Functional Theory (DFT). The exchange and correlations were dealt with the Perdew-Burke Ernzerhof (PBE) functional within the Generalized Gradient Approximation (GGA) framework. The Brillourin zone was sampled with Monkhorst K-point mesh 3 × 2 × 1 through all the computational process. In the differential charge density and density of states calculation process, the structures were geometrically optimized and considered as converged with the energy tolerance less than 10^{-4} eV, iterative calculation using conjugate gradient method. In the work function calculation process, periodic

boundary conditions were used in all directions and a vacuum layer of 15 Å was used in the z direction to separate the slabs.

Supplementary Figures

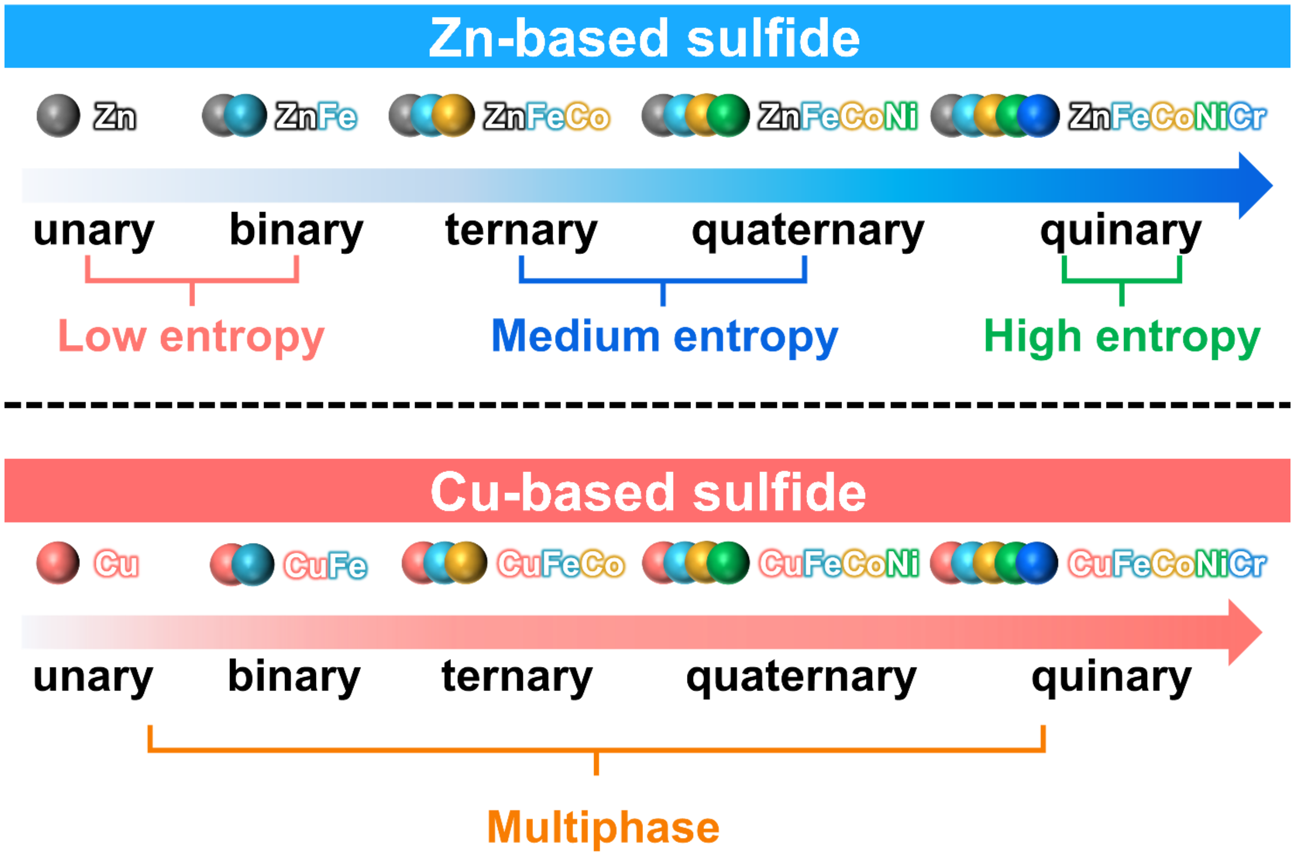

Figure S1. The prepared multi-principal Zn-based/Cu-based sulfides and their subset species.

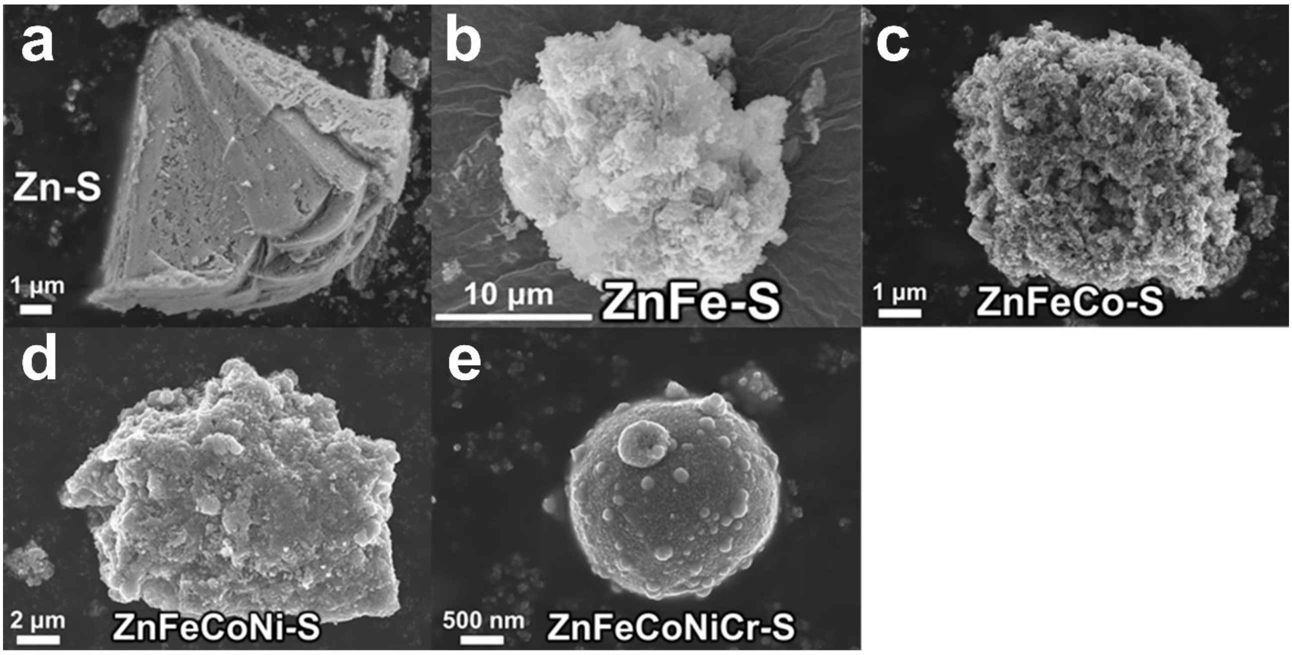

Figure S2. The microscopic morphology of Zn-based sulfides (from unary to quinary). a) Zn-S, b) ZnFe-S, c) ZnFeCo-S, d) ZnFeCoNi-S, e) ZnFeCoNiCr-S.

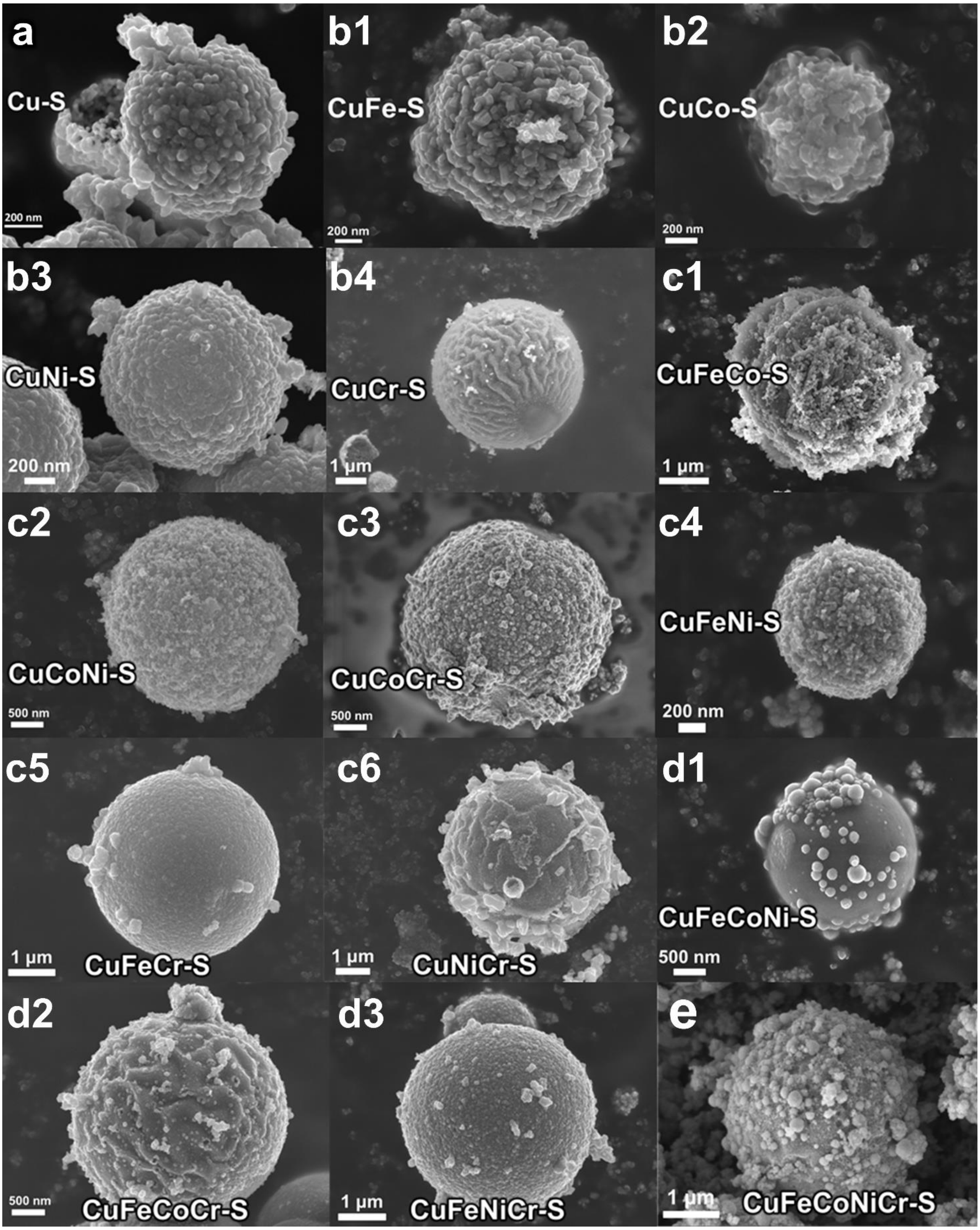

Figure S3. The microscopic morphology of Cu-based sulfides (from unary to quinary). a) unary Cu-based sulfide Cu-S, b1-b4) binary Cu-based sulfides, c1-c5) ternary Cu-based sulfides, d1-d3) quaternary Cu-based sulfides, e) quinary Cu-based sulfide ZnFeCoNiCr-S.

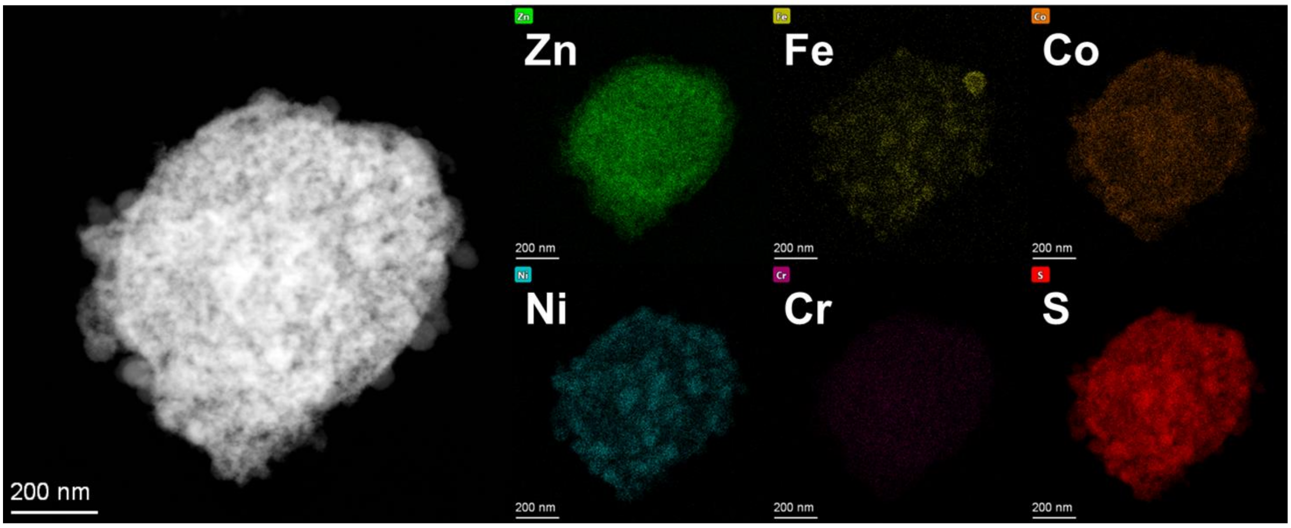

Figure S4. The high angle annular dark field (HAADF) images of high-entropy ZnFeCoNiCr-S sample, indicating the uniform distribution of all elements.

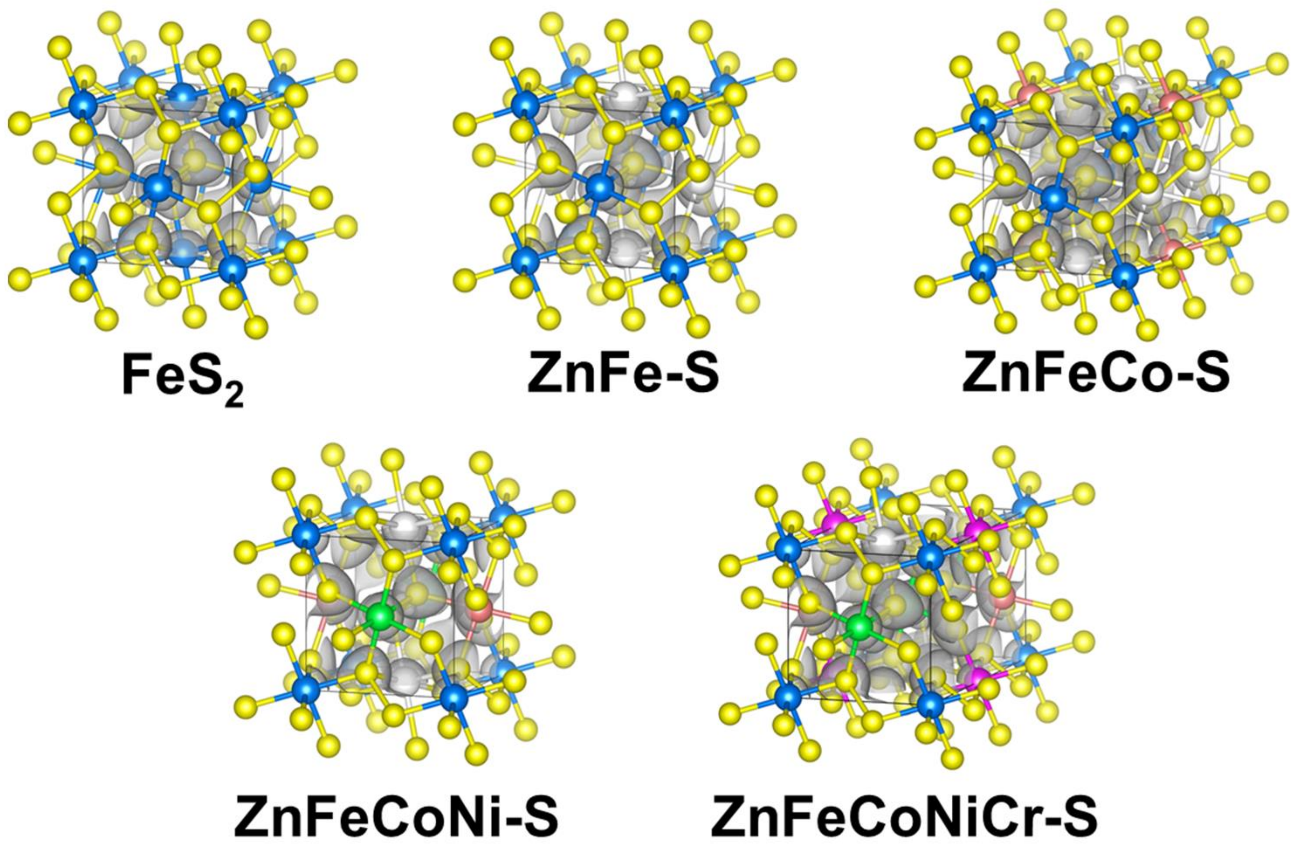

Figure S5. The charge distribution in the FCC structure from unary to quinary.

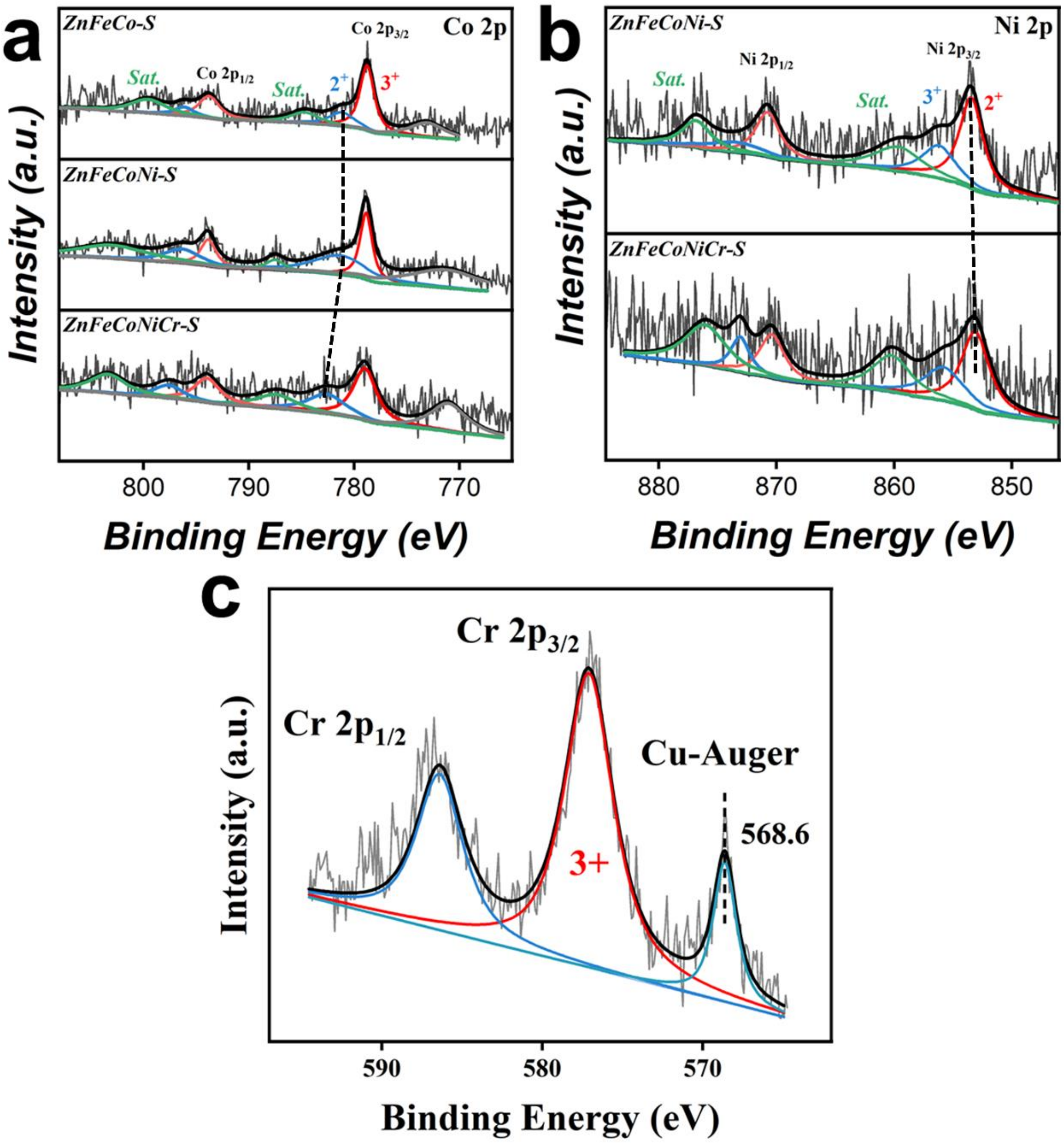

Figure S6. The XPS fitting curves of Co 2p, Ni 2p and Cr 2p orbitals in Zn-based sulfide.

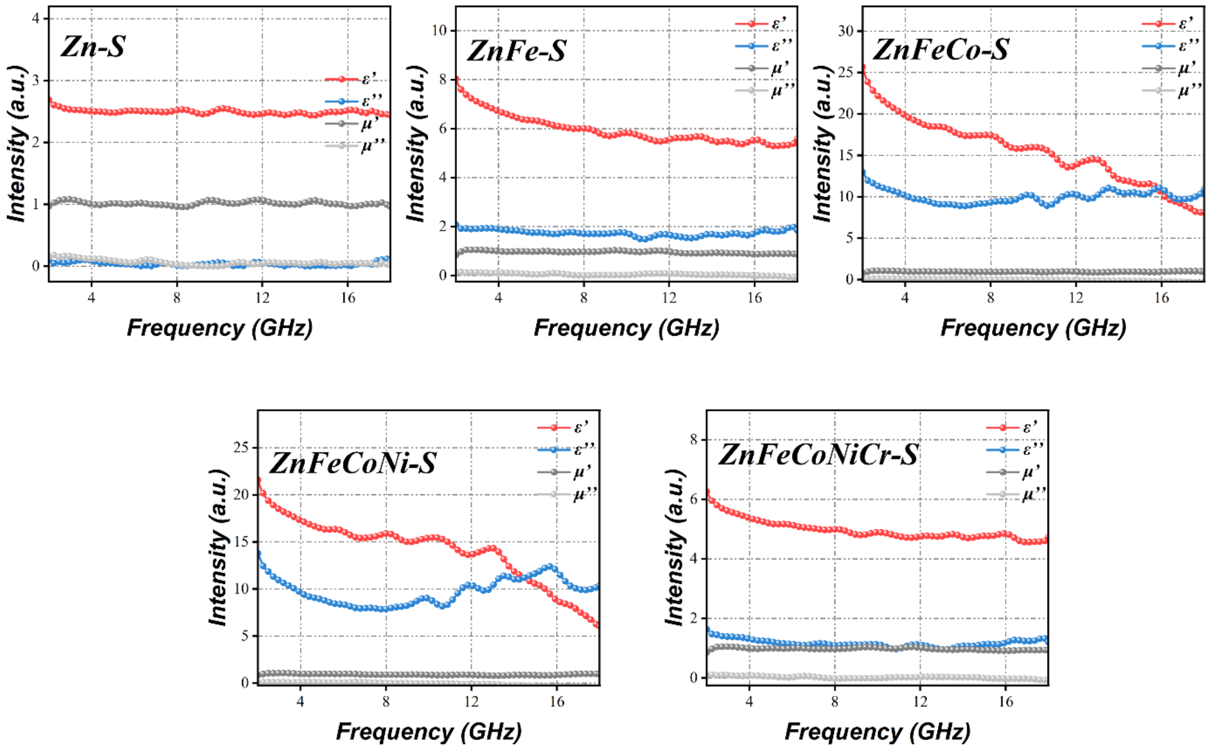

Figure S7. The measured EM parameters of Zn-based sulfides.

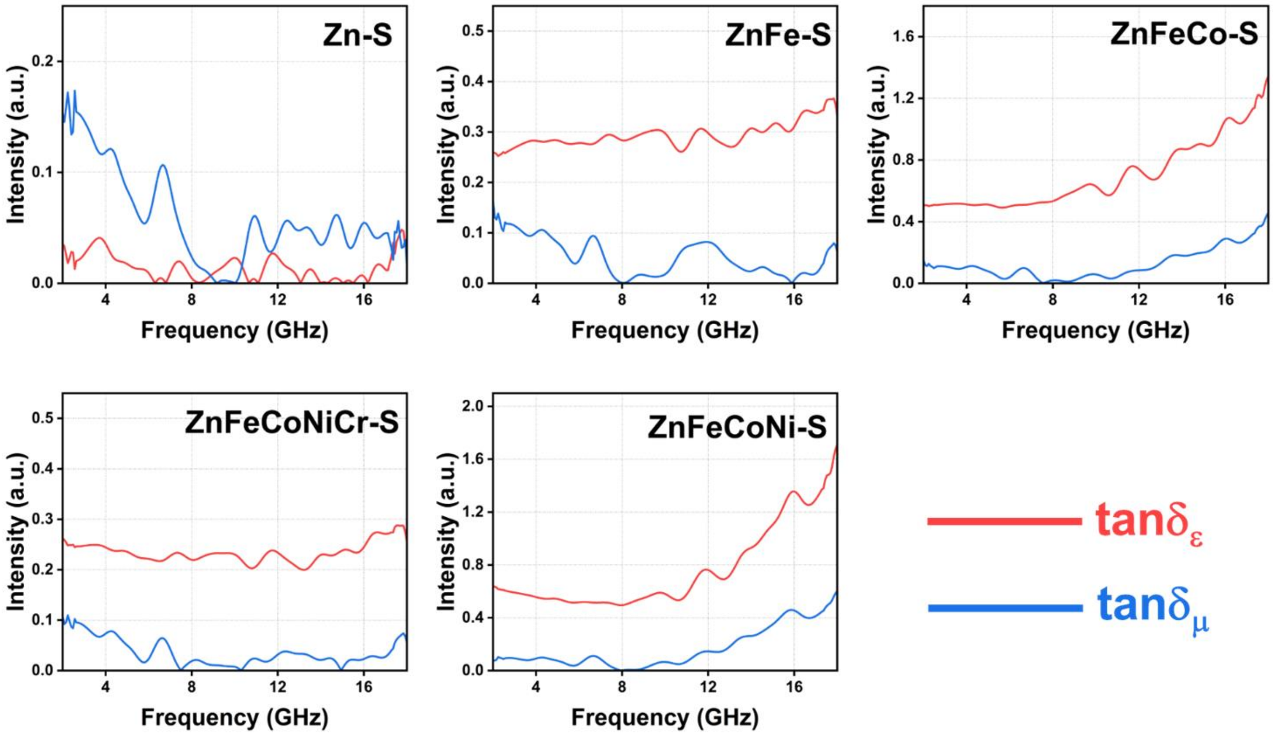

Figure S8. The $\tan\delta_\varepsilon$ and $\tan\delta_\mu$ of Zn-based sulfides.

All of the Zn-based sulfides have $\tan\delta_\varepsilon$ larger than $\tan\delta_\mu$, except for the Zn-S sample. This is because the complex permittivity of the Zn-S sample is too low to effectively dissipate EMW. In this case, it is not meaningful to evaluate its loss mechanism using the loss tangent value.

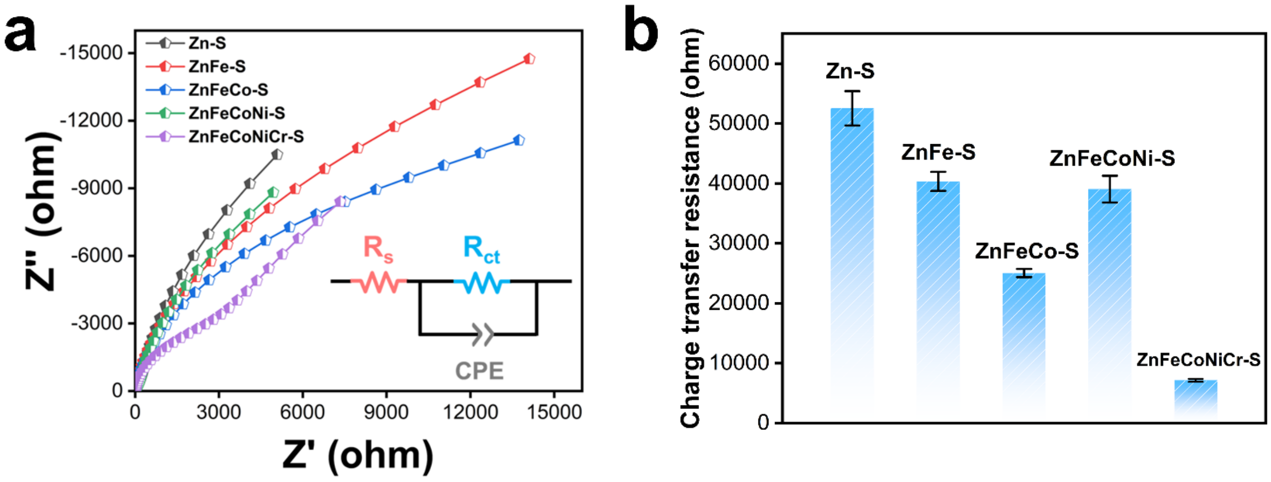

Figure S9. a) The Nyquist curves of Zn-based sulfides. b) The fitting R_{ct} of Zn-based sulfides.

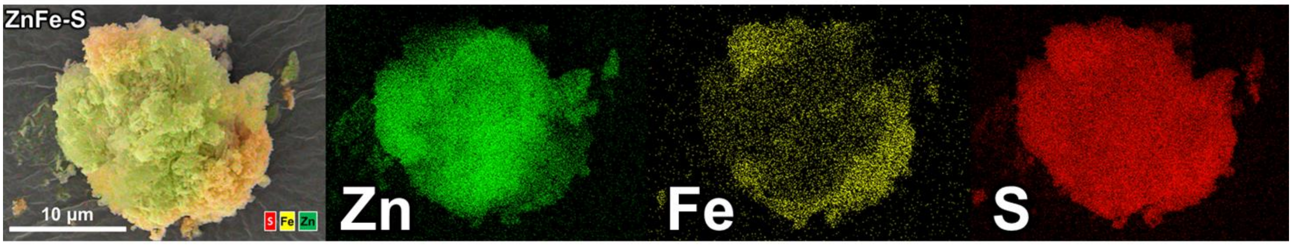

Figure S10. The EDS mapping of ZnFe-S sample, suggesting that significant phase-separation occurs in the ZnFe-S sample.

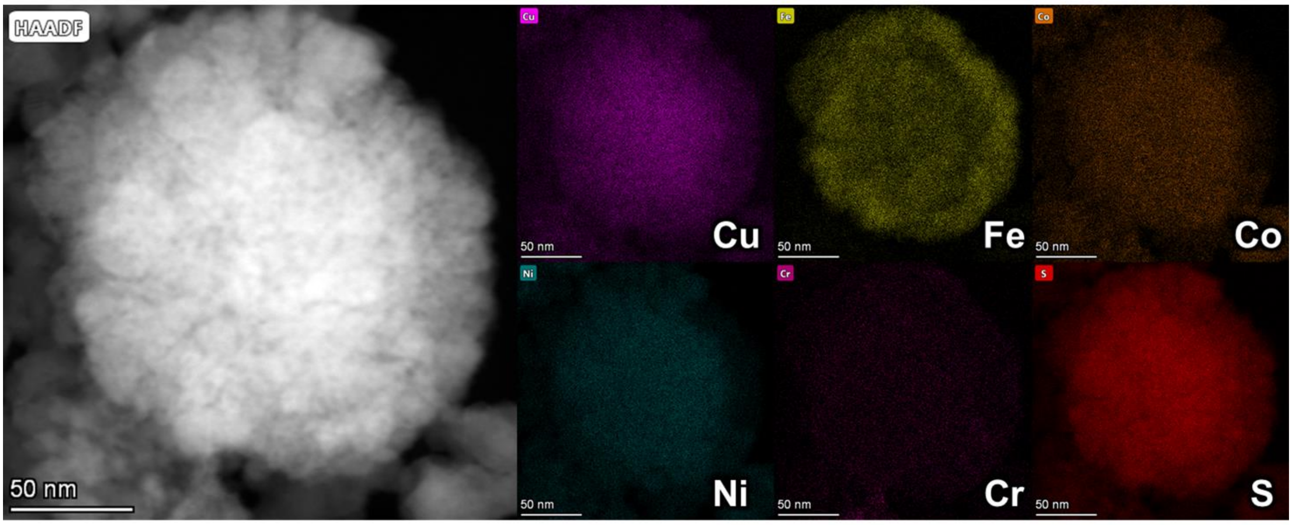

Figure S11. The HAADF images and corresponding element distribution of CuFeCoNiCr-S sample.

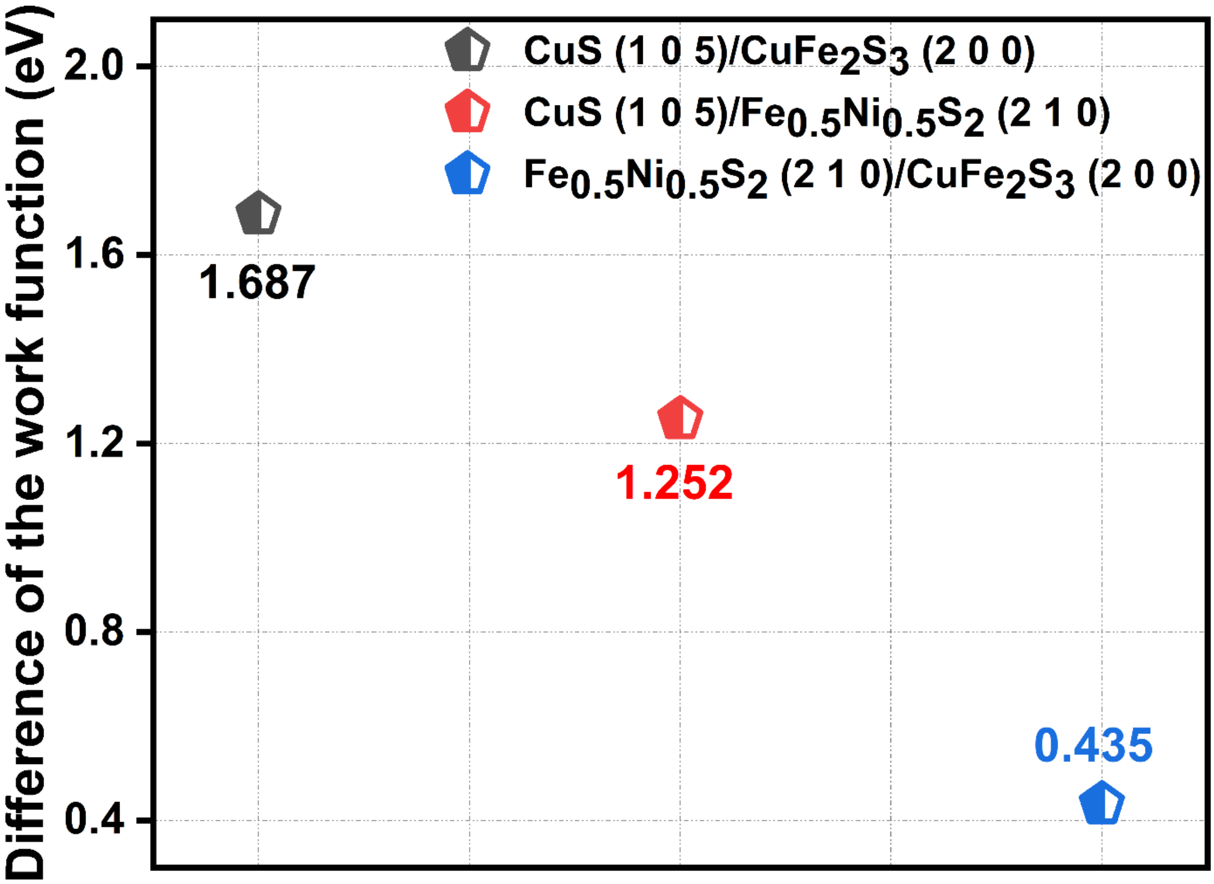

Figure S12. The difference of the work function in Cu-based sulfide, indicating the strength of interfacial polarization. These values are obtained according to unit conversions (**1 Ha≈27.21 eV**).

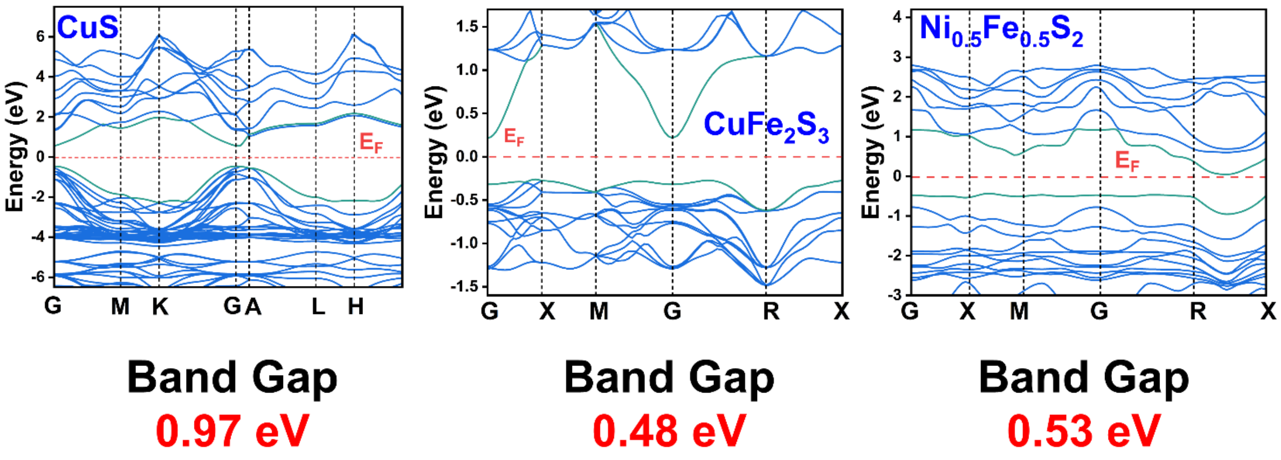

Figure S13. The energy band images of CuS, $CuFe_2S_3$ and $Ni_{0.5}Fe_{0.5}S_2$.

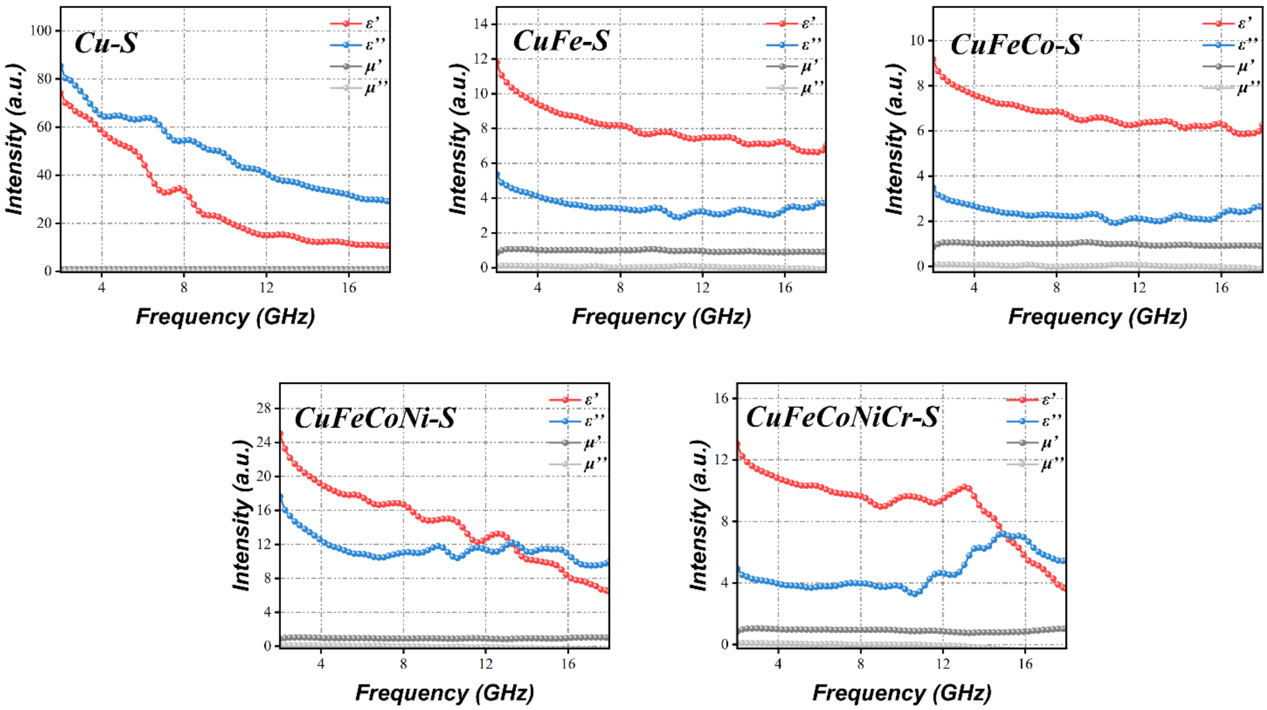

Figure S14. The EM parameters of Cu-based sulfide (including ε′, ε″, μ′ and μ″).

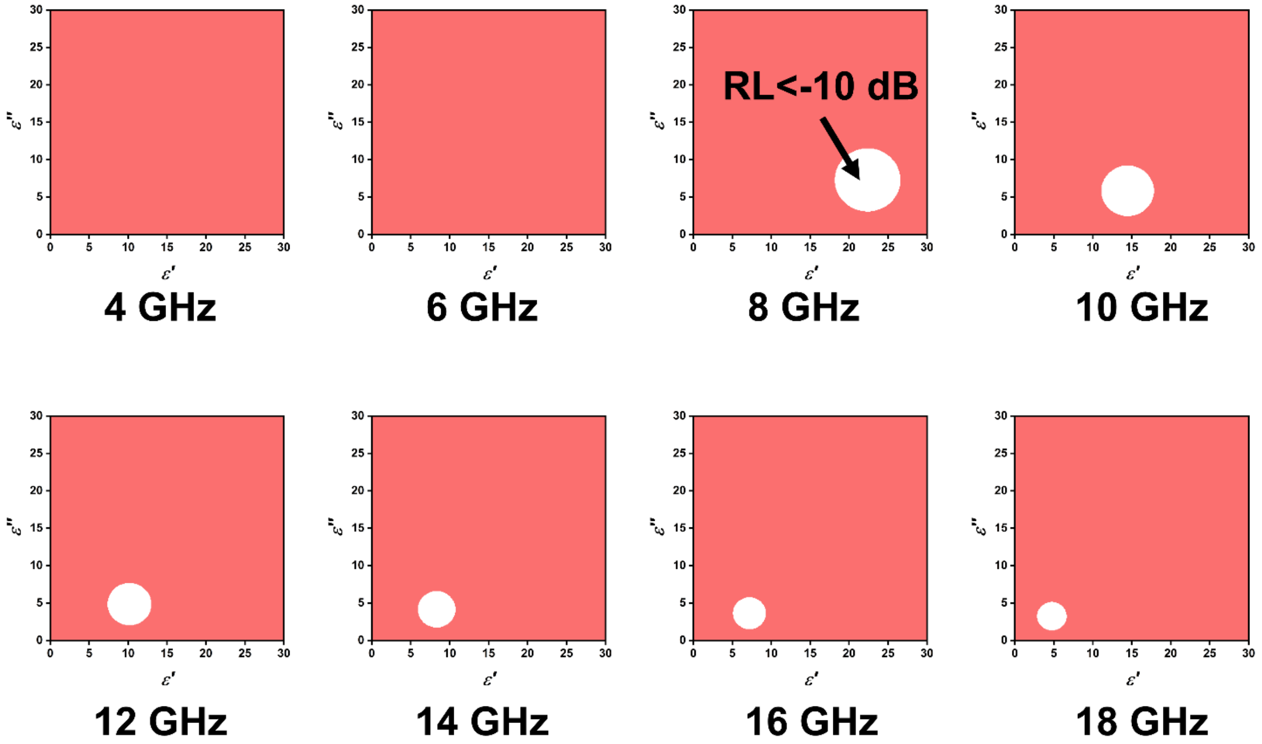

Figure S15. The theoretical prediction range of complex permittivity (ε′ and ε″, white area) for ideal EMW absorption (RL < -10 dB) based on the given parameters (μ′=1, μ″=0 and thickness=2.00 mm).

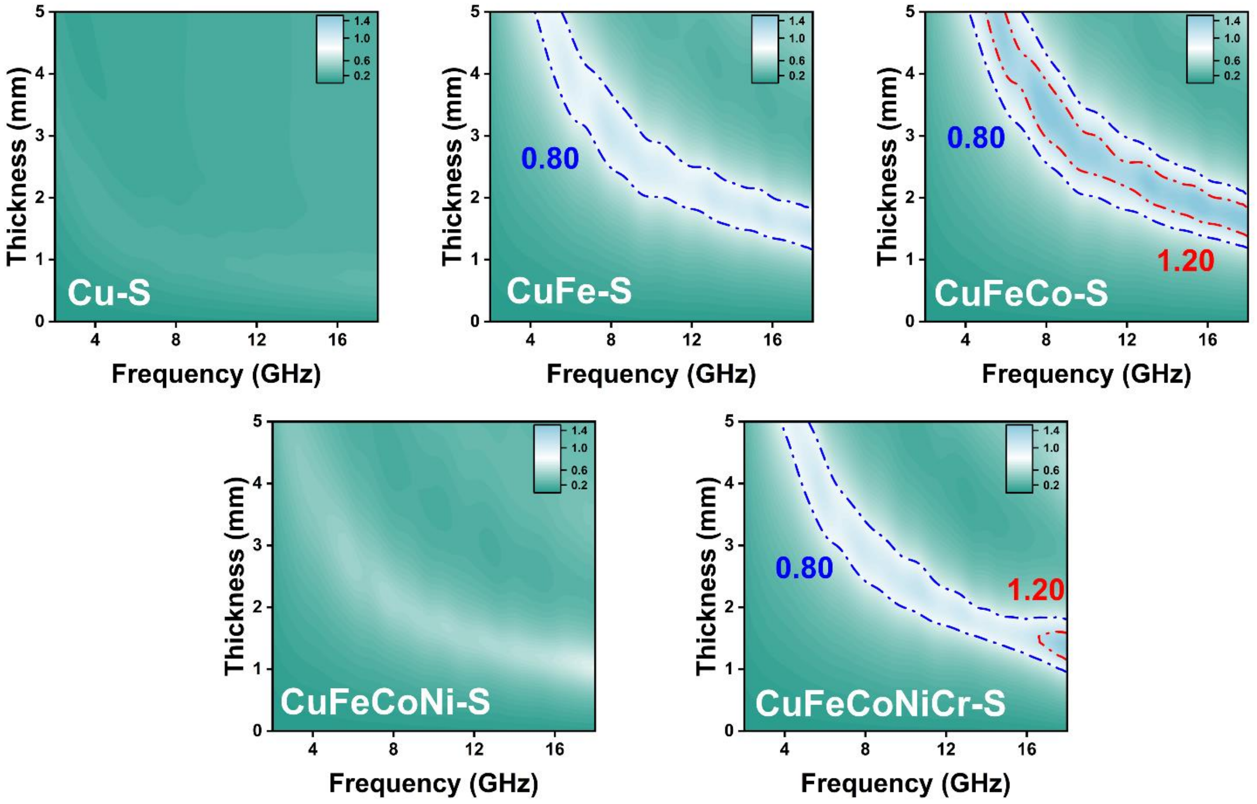

Figure S16. The impedance matching (Z) images of the Cu-based sulfides.

Generally, The larger area between Z = 0.8 (blue lines) and Z = 1.2 (red lines), the better impedance matching level of absorber

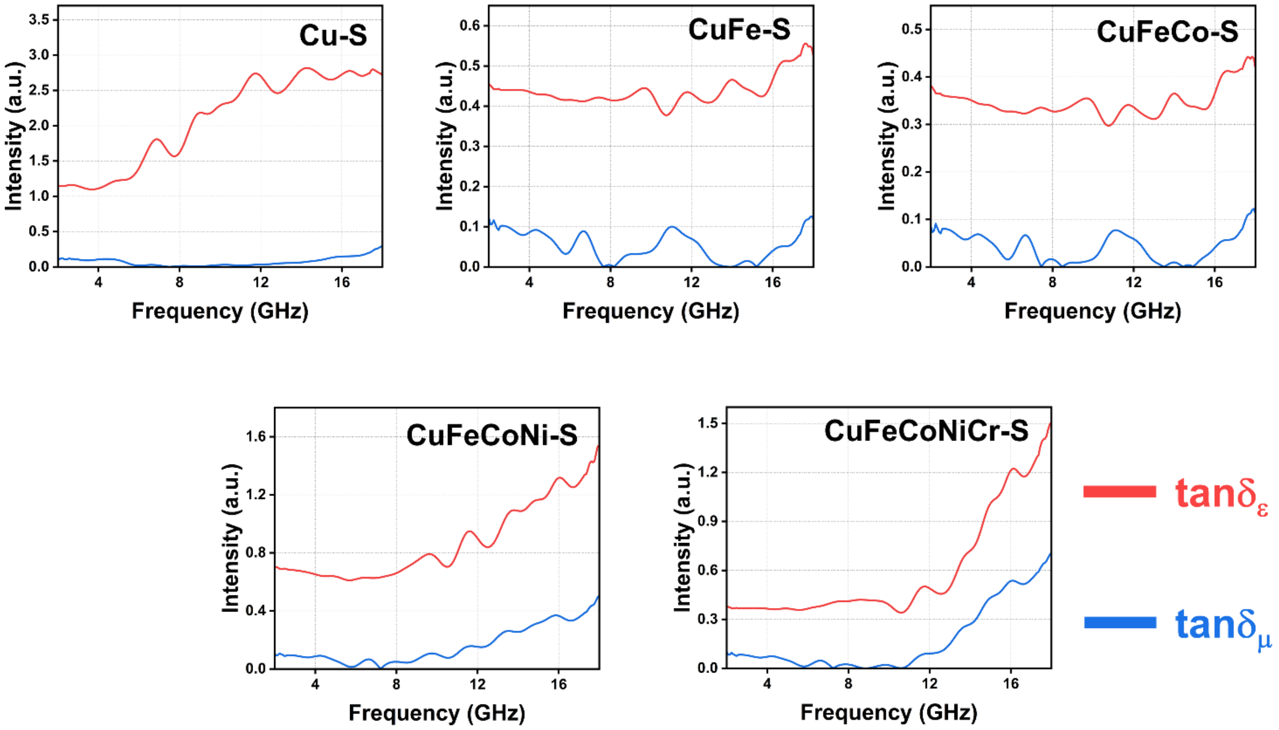

Figure S17. The $\tan\delta_{\varepsilon}$ and $\tan\delta_{\mu}$ of Cu-based sulfides.

All of the Cu-based sulfides have $\tan\delta_{\varepsilon}$ larger than $\tan\delta_{\mu}$, suggesting that these samples are dominated by dielectric loss, while magnetic loss can be neglected.

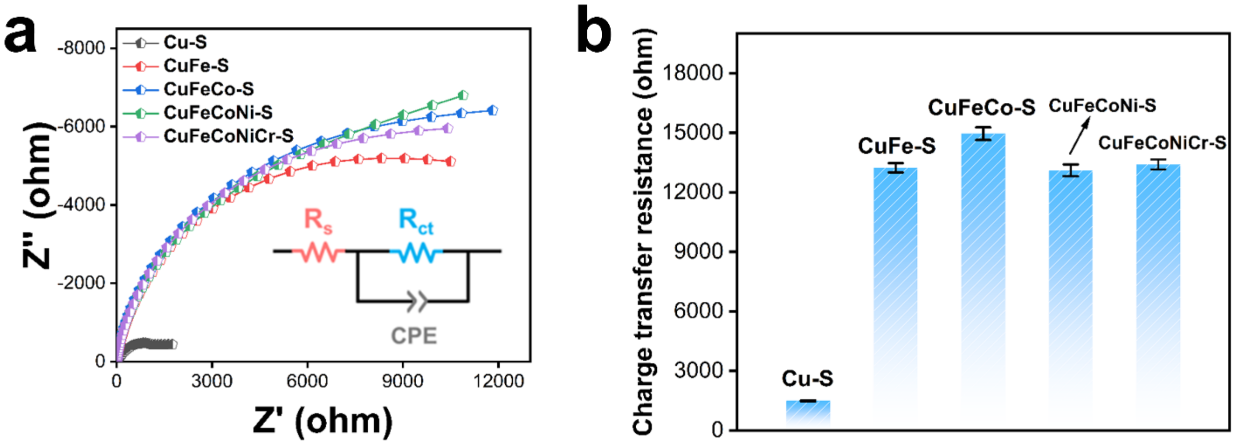

Figure S18. a) The Nyquist curves of Cu-based sulfides. b) The fitting R_{ct} of Cu-based sulfides.

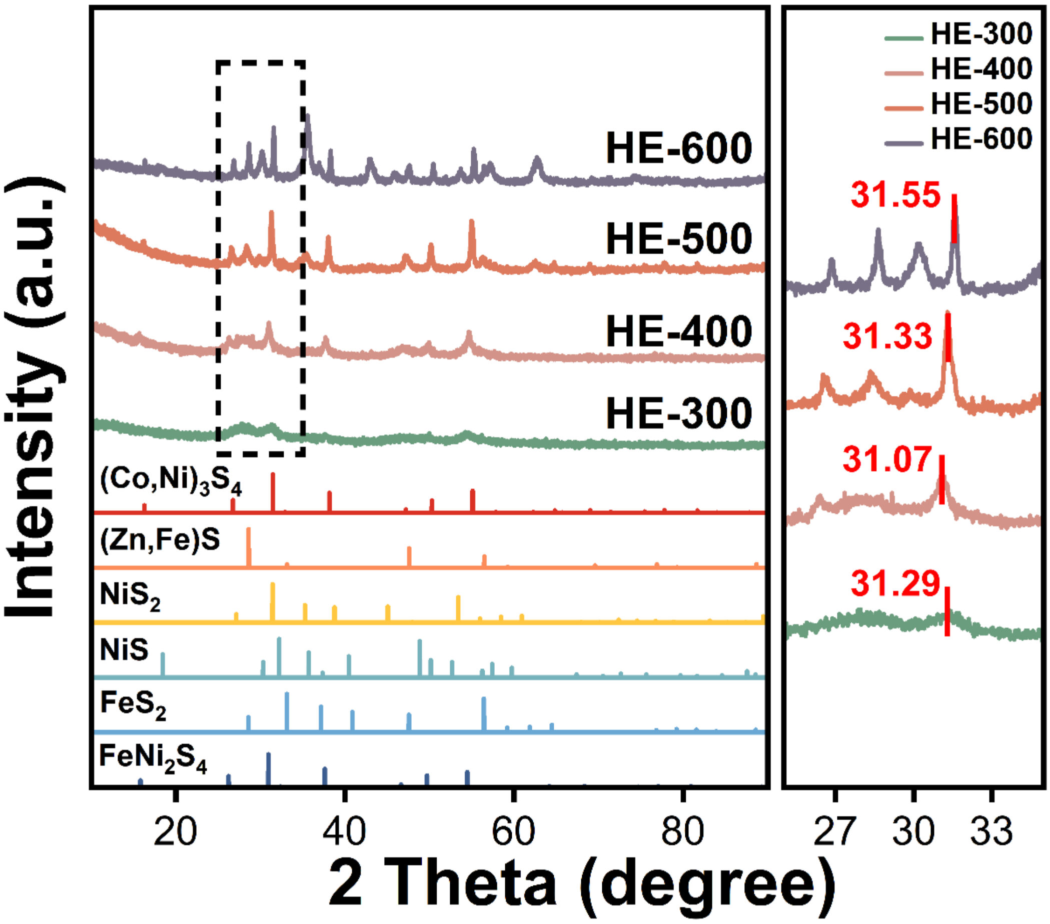

Figure S19. The XRD patterns of HE-300, HE-400, HE-500 and HE-600.

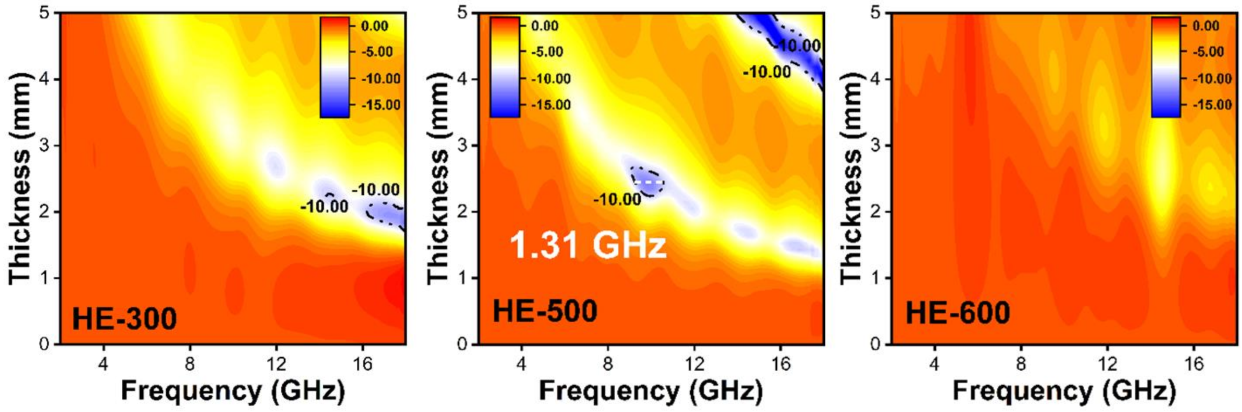

Figure S20. The 2D-RL plots of HE-300, HE-500 and HE-600.

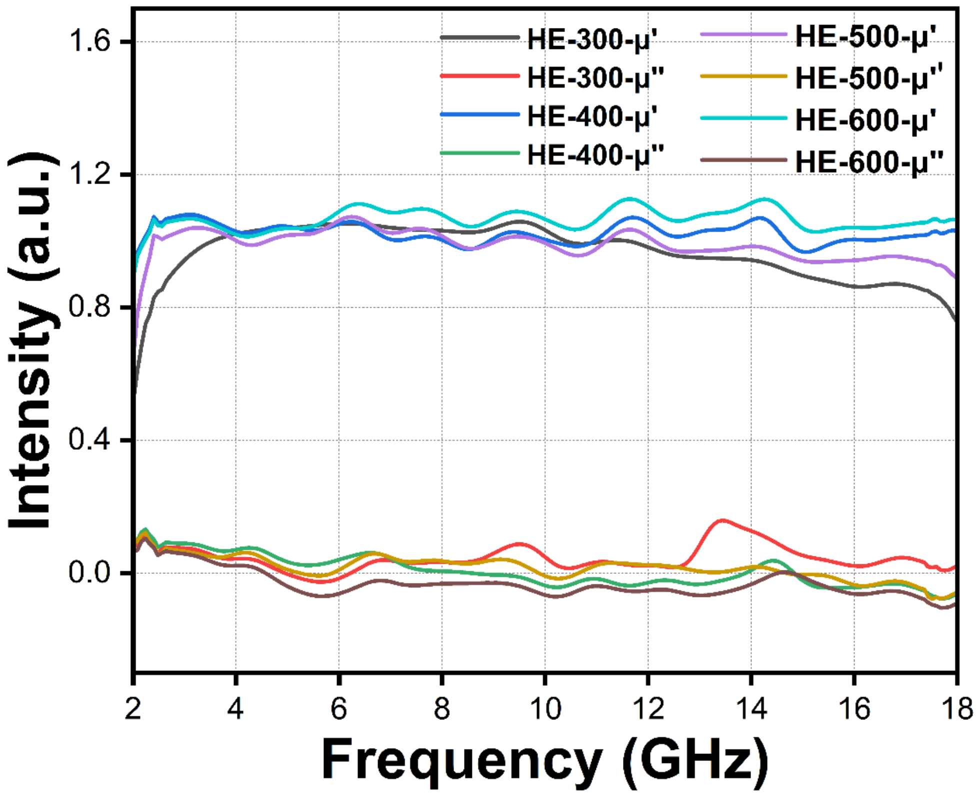

Figure S21. The permeability of HE-300, HE-400, HE-500 and HE-600.

Supplementary Tables

Table S1. The phases of Zn-based sulfides from unary to quinary.

Sample	Phase1	Phase2
Zn-S	ZnS	N/A
ZnFe-S	ZnS	FeS_2
ZnFeCo-S	FeS_2	N/A
ZnFeCoNi-S	FeS_2	N/A
ZnFeCoNiCr-S	FeS_2	N/A

Table S2. The phases of Cu-based sulfides from unary to quinary.

Sample	Phase1	Phase2	Phase3
Cu-S	CuS	$Cu_{1.95}S$	N/A
CuFe-S	CuS	$CuFe_2S_3$	N/A
CuFeCo-S	$Cu_{1.95}S$	$CuFe_2S_3$	N/A
CuFeCoNi-S	CuS	$(Ni_{0.5}Fe_{0.5})S_2$	N/A
CuFeCoNiCr-S	CuS	$CuFe_2S_3$	$(Ni_{0.5}Fe_{0.5})S_2$

Table S3. The EMW absorption properties of Zn-based sulfides.

Sample	EAB (GHz)	Thickness (mm)	RL_{min} (dB)
Zn-S	0	N/A	-2.16
ZnFe-S	**4.24 (The best)**	2.45	-16.88
ZnFeCo-S	1.43	1.27	-11.13
ZnFeCoNi-S	1.74	2.02	-12.20
ZnFeCoNiCr-S	0	N/A	-9.59

Table S4. The EMW absorption properties of Cu-based sulfides.

Sample	EAB (GHz)	Thickness (mm)	RL_{min} (dB)
Cu-S	0	N/A	-3.18
CuFe-S	5.65	2.00	-66.80
CuFeCo-S	0	N/A	-8.98
CuFeCoNi-S	1.23	1.31	-10.49
CuFeCoNiCr-S	**6.70 (The best)**	2.00	-53.15

Table S5. The phases of heat-treated high-entropy ZnFeCoNiCr-S sample in different temperature.

**Sample**	**Phase1**	**Phase2**	**Phase3**	**Phase4**	**Phase5**
HE-300	$(Co, Ni)_3S_4$	(Zn, Fe)S	N/A	N/A	N/A
HE-400	$FeNi_2S_4$	(Zn, Fe)S	N/A	N/A	N/A
HE-500	$(Co, Ni)_3S_4$	(Zn, Fe)S	NiS_2	N/A	N/A
HE-600	$(Co, Ni)_3S_4$	(Zn, Fe)S	NiS_2	NiS	FeS_2

Table S6. The EMW absorption properties of HE-300, HE-400, HE-500 and HE-600.

**Sample**	**EAB (GHz)**	**Thickness (mm)**	**RL_{min} (dB)**
HE-300	1.75	2.00	-15.07
HE-400	**4.83 (The best)**	1.45	-17.26
HE-500	1.31	2.40	-20.00
HE-600	**0**	N/A	-3.11
